# Comparison of Four Commercial Kits for Isolation of Urinary Cell-Free DNA and Sample Storage Conditions

**DOI:** 10.3390/diagnostics10040234

**Published:** 2020-04-18

**Authors:** Eun Young Lee, Eun-Ju Lee, Hana Yoon, Dong Hyeon Lee, Kwang Hyun Kim

**Affiliations:** 1 Department of Urology, College of Medicine, Ewha Womans University, Seoul 07804, Korea; silvero0130@gmail.com (E.Y.L.); ionantha@hanmail.net (E.-J.L.); wowhana@ewha.ac.kr (H.Y.); leedohn@ewha.ac.kr (D.H.L.); 2 Ewha Medical Research Institute, College of Medicine, Ewha Womans University, Seoul 07804, Korea

**Keywords:** urinary cell DNA, liquid biopsy, commercial kit

## Abstract

Urinary cell-free DNA (cfDNA) is an attractive body fluid for liquid biopsy. In this study, we compared the efficiencies of four commercial kits for urinary cell-free DNA (cfDNA) isolation and of various sample storage conditions. Urinary cfDNA was isolated from 10 healthy individuals using four commercial kits: QIAamp Circulating Nucleic Acid Kit (QC; Qiagen), MagMAX™ Cell-Free DNA Isolation Kit (MM; Applied Biosystems), Urine Cell-Free Circulating DNA Purification Midi Kit (NU; Norgen Biotek), and Quick-DNA™ Urine Kit (ZQ; Zymo Research). To assess the isolation efficiency, an Agilent 2100 Bioanalyzer with High Sensitivity DNA chips was used, and cfDNA yield was defined as the amount of cfDNA obtained from 1 mL of urine. MM and QC provided the highest cfDNA yield in the 50–300 bp range, and MM and NU gave the highest cfDNA yield in the 50–100 bp range. In particular, the NU kit was efficient for isolation of more fragmented cfDNA in the range of 50–100 bp with the lowest cellular genomic DNA contamination. ZQ had the best cost-efficiency for isolating the same amount of urinary cfDNA. Samples stored at −70 °C with the addition of 10 mM EDTA resulted in the highest cfDNA yield 3 months after sample collection.

## 1. Introduction

Liquid biopsy has emerged as an important non-invasive tool for cancer diagnostics. Liquid biopsy utilizes genetic material from body fluids; this method can overcome the limitations associated with traditional tissue biopsy such as invasiveness and the difficulty of repeated sampling [1,2,3]. Cell-free DNA (cfDNA) is one of the most important sources for liquid biopsy. Genetic analysis of cfDNA allows clinicians to infer the characteristics of a tumor. Many researchers have examined genetic alteration of cfDNA using various analytical tools such as real-time PCR, droplet digital PCR (ddPCR), and next-generation sequencing (NGS) [4,5,6]. To achieve proper results with various genetic tests, it is important to establish a method to extract a high yield of cfDNA. Many researchers have investigated the optimal conditions for each step, encompassing sample collection, handling, and storage to maximize the recovery of cfDNA [7,8,9,10].

Although circulating cfDNA in blood is most widely used in liquid biopsy for malignant disease, cfDNA can also be isolated from various body fluids. Urine is an ideal body fluid for liquid biopsy as it can be collected in a truly non-invasive manner with a relatively reduced limit in volume. Specifically, urine could be more useful in liquid biopsy for urologic malignant disease. Several studies have demonstrated that urinary cfDNA can be utilized as an important source for liquid biopsy in urologic malignant disease [5,11,12,13,14]. Due to the increased rate of necrosis of tumor cells, urinary cfDNA carries a higher tumor genomic burden than cellular DNA [11,15]. In addition, urinary cfDNA also reflects the systemic status of patients, as it is derived from both the urinary tract and the circulatory system [16]. However, although many studies have compared the various extraction methods of circulating cfDNA in blood [17,18,19], limited data are available on methods for urinary cfDNA extraction [20,21]. Most studies have been conducted on urinary DNA, regardless of being genomic DNA or cfDNA [22,23,24].

In this study, we aimed to compare the urinary cfDNA isolation efficiency of four commercial kits with samples from healthy individuals. The four commercial kits were QIAamp Circulating Nucleic Acid Kit (QC; Qiagen, Hilden, Germany), MagMAX™ Cell-Free DNA Isolation Kit (MM; Applied Biosystems, Thermo Fisher Scientific, Foster City, CA, USA), Urine Cell-Free Circulating DNA Purification Midi Kit (NU; Norgen Biotek, Thorold, ON, Canada), and Quick-DNA™ Urine Kit (ZQ; Zymo Research, Irvine, CA, USA). Isolation efficiency was assessed based on the level of urinary cfDNA yield and genomic DNA contamination in an electropherogram. We also compared four different sample storage conditions according to temperature and preservative.

## 2. Materials and Methods

### 2.1. Sample Collection and Storage

**Figure 1:**
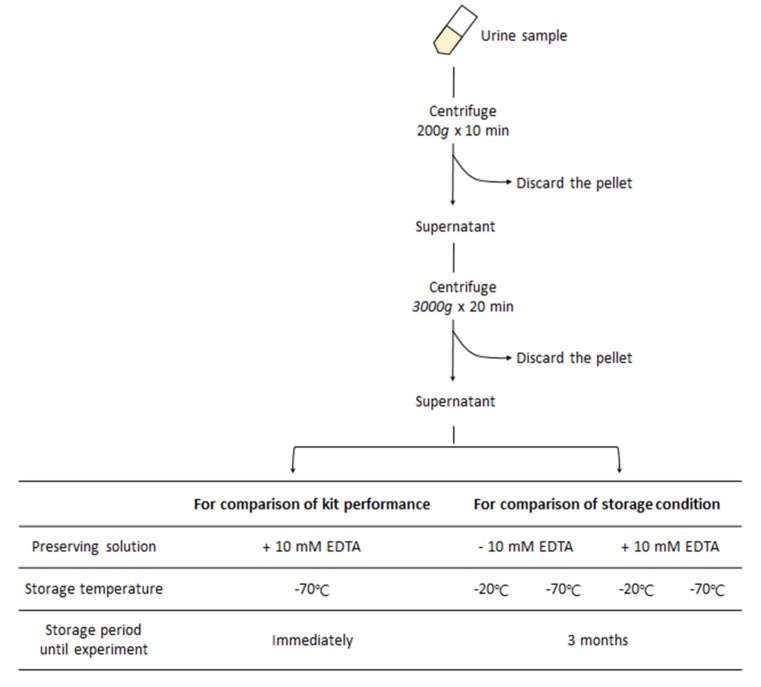
Sample preprocessing.

Urine samples were obtained from five healthy males and five healthy females. The mean ages of males and females were 39.6 (range 34–45) and 33.8 (range 26–45), respectively. This study was approved by the Institutional Review Board of Ewha Medical Center (IRB No. 2018-08-027-004) and all participants gave their informed consent in writing. The first morning, urine was collected and the urine samples were centrifuged at 200 *g* for 10 min followed by 3000 *g* for 20 min to remove cellular material. The supernatants after two centrifugations were mixed with or without 10 mM EDTA and stored at −20 or at −70 °C until used for experiments (Figure 1). 

### 2.2. cfDNA Isolation

We compared the isolation efficiency of four commonly used commercial kits: QIAamp Circulating Nucleic Acid Kit (QC; Qiagen, Hilden, Germany), MagMAX™ Cell-Free DNA Isolation Kit (MM; Applied Biosystems, Thermo Fisher Scientific, Foster City, CA, USA), Urine Cell-Free Circulating DNA Purification Midi Kit (NU; Norgen Biotek, Thorold, ON, Canada), and Quick-DNA™ Urine Kit (ZQ; Zymo Research, Irvine, CA, USA). The frozen urine samples were thawed at 4 °C and centrifuged again at 3000 *g* for 10 min to remove impurities in the samples. The starting volume was determined according to the manufacturer’s recommendation of each kit; 4, 4, 10, and 24 mL of urine were used for QC, MM, NU, and ZQ, respectively. 

### 2.3. Analysis of DNA Fragments Using a Bioanalyzer

**Figure 2:**
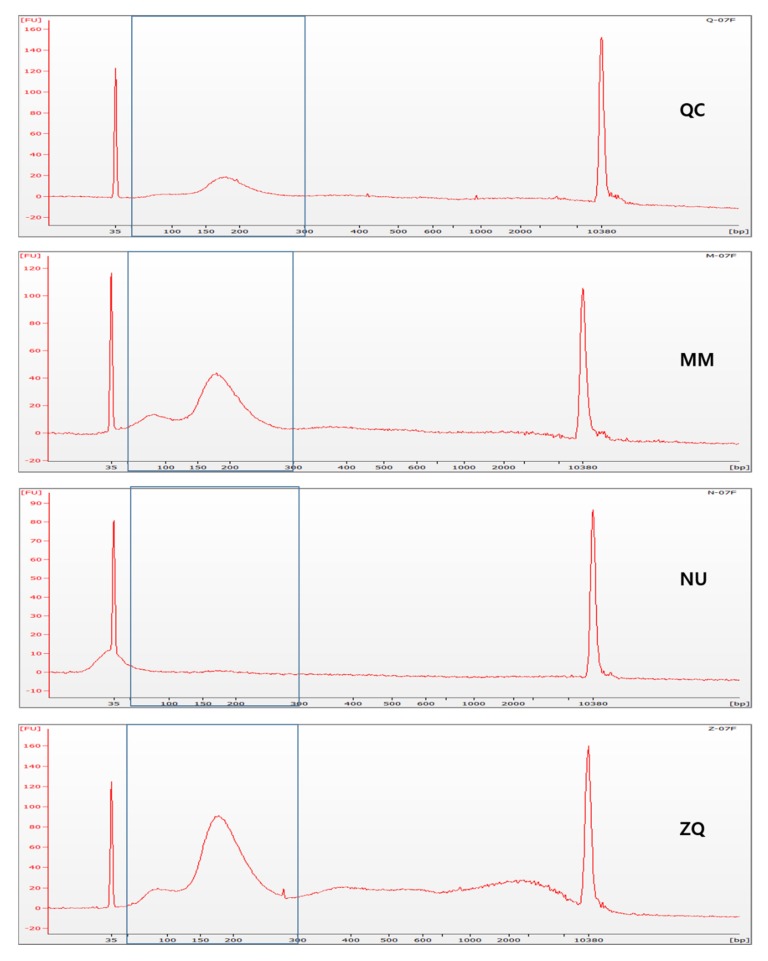
Representative electropherograms visualized on the Bioanalyzer using high sensitivity DNA chips. Electropherograms show the cell-free DNA (cfDNA) fragment distributions obtained by four different extraction methods using the same sample. The cfDNA concentration was calculated for fractions in the size range of 50–300 bp (QC; QIAamp Circulating Nucleic Acid Kit, MM; MagMAX™ Cell-Free DNA Isolation Kit, NU; Norgen Urine Cell-Free Circulating DNA Purification Midi Kit, ZQ; Zymo research Quick-DNA™ Urine Kit).

To assess DNA fragment distribution and concentration, cfDNA was electrophoresed on an Agilent 2100 Bioanalyzer with High Sensitivity DNA chips (Agilent technologies Inc., Santa Clara, CA, USA). The cfDNA concentration was measured from the defined range of 50–300 bp on electropherograms (Figure 2). The cfDNA isolation efficiency of four commercial kits was compared by cfDNA yield, which was defined as the amount of cfDNA obtained from 1 mL of urine. As urinary cfDNA is known to be more fragmented [25,26,27], we also measured the cfDNA concentration in the range of 50–100 bp on electropherograms. To estimate the level of contamination by cellular genomic DNA, the concentration of high molecular weight (HMW) DNA (>1 kb) was measured, and the ratio of 50–300 bp cfDNA to HMW DNA was calculated.

### 2.4. Comparison of Sample Storage Conditions

**Figure 3:**
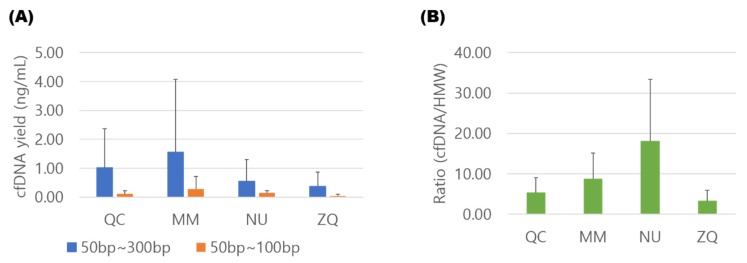
Comparison of cfDNA isolation efficiencies by four different extraction methods. (**A**) Comparison of the cfDNA yield expressed as the amount of DNA obtained from 1 mL of urine. (**B**) Comparison of the degree of contamination by cellular genomic DNA. The degree of contamination was assessed as the ratio of cfDNA (50–300 bp) to high molecular weight (HMW) DNA (>1 kb) (QC; QIAamp Circulating Nucleic Acid Kit, MM; MagMAX™ Cell-Free DNA Isolation Kit, NU; Norgen Urine Cell-Free Circulating DNA Purification Midi Kit, ZQ; Zymo research Quick-DNA™ Urine Kit).

To compare different sample storage conditions for minimizing cfDNA loss, urine supernatant from 10 healthy individuals was aliquoted into four tubes and kept under four different conditions: (1) storage at −20 °C with the addition of 10 mM EDTA, (2) storage at −20 °C without the addition of EDTA, (3) storage at −70 °C with the addition of 10 mM EDTA, and (4) storage at −70 °C without the addition of EDTA (Figure 3). EDTA was used as the preservative as it could be easily used to inactivate nuclease activity [13]. After three months, the frozen sample was thawed at 4 °C and centrifuged at 3000 *g* for 10 min and was then subjected to cfDNA isolation. The average cfDNA yield of 10 samples was compared according to each storage condition. 

## 3. Results

### 3.1. Comparison of cfDNA Isolation Efficiency

The cfDNA was isolated immediately after sample collection using the four commercial kits, and cfDNA concentration was analyzed using electropherograms from an Agilent 2100 Bioanalyzer with High Sensitivity DNA chips (Agilent technologies Inc., Santa Clara, CA, USA). The cfDNA yield was calculated as the amount of cfDNA from 1 mL of urine. In the range of 50–300 bp, MM and QC exhibited the highest average cfDNA yield. Of 10 samples, five (50%) and four (40%) showed the highest cfDNA yield by MM and QC, respectively. For more fragmented cfDNA in the range of 50–100 bp, the MM and NU showed the highest average cfDNA yield. MM and NU showed the highest cfDNA yield in four (40%) and six samples (60%), respectively (Figure 3A) (Supplementary Table S1). 

### 3.2. Contamination of Cellular Genomic DNA

DNA fragments in the region of over 1000 bp were considered to be derived from urinary cellular DNA [13], and the DNA concentration of high molecular weight (HMW) DNA above 1 kb was measured to examine the degree of contamination by cellular DNA. Figure 3B shows the yield ratio of 50–300 bp cfDNA to HMW DNA obtained from 1 mL of urine. Although MM showed the highest cfDNA yield, an average ratio of cfDNA to HMW DNA was highest with NU, indicating that NU isolated cfDNA of high purity with low cellular genomic DNA contamination. Of 10 samples, six (60%) and three (30%) showed the highest ratio of cfDNA to HMW DNA by NU and MM, respectively (Supplementary Table S2).

### 3.3. Comparison of Storage Conditions for Urinary cfDNA

**Figure 4:**
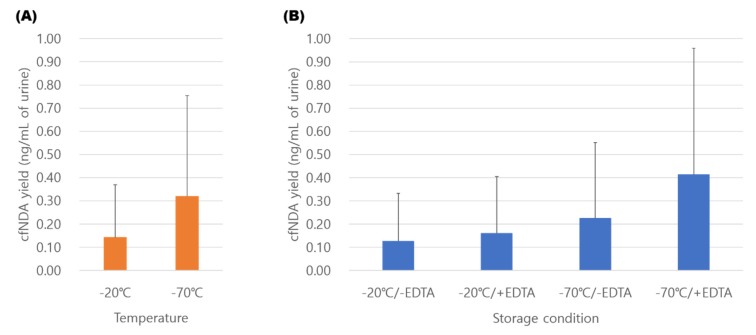
Comparison of cfDNA isolation yields from four different storage conditions. The yield of cfDNA was expressed as the amount of DNA obtained from 1 mL of urine. Values shown in the graph are averages calculated within each storage condition group. (**A**) The average yield within the same temperature condition. (**B**) The average yield for four different storage conditions.

To compare the effect of storage conditions on urinary cfDNA extraction, urine samples were stored under four different conditions for 3 months (Figure 1). As the MM kit showed the highest average cfDNA yield in the range of 50–300 bp, cfDNA isolation was conducted using MM. The cfDNA yields from samples stored at −70 °C were higher than those stored at −20 °C. At each storage temperature condition, samples with 10 mM EDTA preservative resulted in higher yields of cfDNA (Figure 4). Of 10 samples, six (60%) had the highest cfDNA yield at a storage condition of −70°C in 10 mM EDTA. Our results demonstrate that urine stored at −70 °C with 10 mM EDTA minimizes cfDNA loss. Of 10 samples stored at −70 °C with 10 mM EDTA, five samples maintained a cfDNA yield of greater than 80%, but four samples maintained less than 20% of the cfDNA yield compared to fresh urine samples (Supplementary Table S3).

## 4. Discussion

In this study, we compared four commonly used commercial kits for urinary cfDNA isolation and our results show that each kit has its own characteristics and advantages. We summarize the characteristics of each kit in Table 1. QC specializes in cfDNA extraction, and many researchers have reported its efficient purification performance [17,18,19]. The QC has also been widely used for liquid biopsy using urinary cfDNA in various urologic and non-urologic malignancies [5,28,29,30]. The MM utilizes a magnetic bead-based extraction method. Magnetic bead-based methods have been developed in many forms to extract DNA with high purity [24,31]. Moreover, high molecular weight DNA and low molecular weight DNA can be separated from the entire DNA and recovered using magnetic beads [31,32]. In our previous study, urinary cfDNA was efficiently isolated with MM in patients with urinary bladder cancer and subsequent sequencing was successfully performed [12]. Our results show high efficiency for both QC and MM for urinary cfDNA isolation. In addition, both methods can be automated, providing efficiency when handling a large quantity of samples. Compared to QC and MM, NU shows different features with a higher yield in the size distribution 50–100 bp. Although MM had a higher average yield than NU, 60% (6/10) of samples had the highest yield with NU in the range of 50–100 bp. In addition, the level of cellular DNA contamination was relatively low, indicating that NU could be preferentially used for extraction of small fragmented cfDNA with high purity. ZQ shows relatively low urinary cfDNA yield compared to other commercial kits. However, ZQ has the largest processing volume and cfDNA can be isolated from up to 40 mL of urine per standard preparation. In terms of cost-effectiveness, the ZQ kit has advantages over the other commercial kits. To extract 1 ng of cfDNA from urine with our protocol costs $2.7 with ZQ, while QC and MM cost $7.6 and $5.0, respectively. ZQ has been widely used for urinary cfDNA isolation in multiple studies of liquid biopsies for urologic malignancies [11,14,33]. In fact, ZQ is designed for both cfDNA and cellular DNA. For sequencing library preparation, size selection using beads would be necessary to remove large DNA fragments.

Although it is recommended that cfDNA be extracted from urine as soon as possible, it is inevitable that samples would be stored for extended time in a clinical setting. Therefore, it is important to find the best conditions to maintain cfDNA stability during long-term storage. Urine has a stronger activity of DNase I than tissues or other body fluids [34], thus it is crucial to inhibit the DNase activity primarily. As shown in our results, treatment with EDTA, a known chelating agent, is effective in inhibiting cfDNA degradation. EDTA is commonly used as a preservative for urine storage and for the protection of DNA, including genomic DNA and cfDNA [35,36]. With regard to storage temperature, the cfDNA stored at −70 °C remained more stable than at −20 °C in line with previous studies that examined urinary genomic DNA or plasma cfDNA [8,35]. Our results suggest that urine should be stored at −70 °C or below with the addition of EDTA for long-term storage. However, the loss of cfDNA was greater than 80% even under the best storage conditions 3 months after collection, indicating that cfDNA extraction should be performed as soon as possible.

For quantification of cfDNA, real-time PCR has been most widely used targeting specific DNA sequences. Recently, various quantification methods such as spectrophotometry or fluorometry have been used for cfDNA quantification. In this study, we used electrophoresis-based instruments (Agilent 2100 Bioanalyzer) which enabled both fluorometric quantification and cfDNA sizing. Compared to spectrophotometry, the fluorometric assay is the preferred method for quantification of cfDNA as spectrophotometry has low sensitivity for analyzing low concentrations of cfDNA [37]. However, fluorometry can overestimate cfDNA concentration as it does not differentiate HMW DNA fragments [38]. Although previous studies have demonstrated the correlation of fluorometric quantification and real-time PCR [39,40], an electrophoresis-based instrument provides a better method considering that genomic DNA contamination is often observed after cfDNA isolation. In addition, urinary cfDNA is known to be more fragmented and variably sized compared to plasma [25,41]. Although real-time PCR is considered the gold standard for DNA quantification, cfDNA fragments smaller than the size of the target amplicon cannot be quantified. In our study, electropherograms of urinary cfDNA show wide shaped peaks around 50–300 bp compared with electropherograms of plasma cfDNA, which usually show sharp peaks around 150 bp. In addition, electropherograms show that variable sizes of DNA were present in the cfDNA extract. In light of characteristics shown in electropherograms, we defined the cfDNA yield based on the size of 50–300 bp. For quantification of more fragmented cfDNA in urine, we additionally measured cfDNA with the size of 50–100 bp.

In summary, we found that the four commercial kits each have advantages. Both MM and QC were efficient in the isolation of urinary cfDNA. For isolation of more fragmented cfDNA, NU could be the choice of method. ZQ permits the largest urine starting volume with the lowest cost. Considering that urinary cfDNA yield is relatively low, ZQ could also be the method of choice if a large volume of sample is available. In terms of storage conditions, −70 °C storage is preferred to −20 °C and the addition of EDTA reduced the loss of urinary cfDNA.

## Figures and Tables

**Table 1:** Summarization of urinary cfDNA isolation kits used in this study.

	Qiagen	Applied Bio Systems	Norgen	Zymo Research
Product name (Abbreviation)	QIAamp Circulating Nucleic Acid Kit (QC)	MagMAX cell-free DNA isolation kit (MM)	Urine cell-free circulating DNA purification kit-midi (NU)	Quick-DNA ™ Urine Kit (ZQ)
Method	Column	Bead	Column	Bead + column
Time for run	1.5 h	2 h	2 h	2 h
Starting volume	4 mL	4 mL	10 mL	24 mL *
Cost for sample	$31	$31	$23	$12
Cost for 1 ng urinary cfDNA isolation †	$7.6	$5.0	$4.1	$2.7

* ZQ allows up to 40 mL of starting volume per standard preparation. In this study, urinary cfDNA was isolated from 24 mL of urine with ZQ. † Cost for 1 ng urinary cfDNA isolation was calculated based on our results (50–300 bp size).

## Supplementary Materials

The following are available online at https://www.mdpi.com/2075-4418/10/4/234/s1.
